# Genome-Wide Association and Genomic Selection for Resistance to Amoebic Gill Disease in Atlantic Salmon

**DOI:** 10.1534/g3.118.200075

**Published:** 2018-02-02

**Authors:** Diego Robledo, Oswald Matika, Alastair Hamilton, Ross D. Houston

**Affiliations:** * The Roslin Institute and Royal (Dick) School of Veterinary Studies and; † Landcatch Natural Selection Ltd., Roslin Innovation Centre, University of Edinburgh, EH25 9RG Midlothian, United Kingdom,and; ‡ Hendrix Genetics Aquaculture BV/ Netherlands, Villa ‘de Körver’, Spoorstraat 69, 5831 CK Boxmeer, The Netherlands

**Keywords:** aquaculture, amoebic gill disease, Salmo salar, disease resistance, animal breeding, fish, GenPred, Shared Data Resources, Genomic Selection

## Abstract

Amoebic gill disease (AGD) is one of the largest threats to salmon aquaculture, causing serious economic and animal welfare burden. Treatments can be expensive and environmentally damaging, hence the need for alternative strategies. Breeding for disease resistance can contribute to prevention and control of AGD, providing long-term cumulative benefits in selected stocks. The use of genomic selection can expedite selection for disease resistance due to improved accuracy compared to pedigree-based approaches. The aim of this work was to quantify and characterize genetic variation in AGD resistance in salmon, the genetic architecture of the trait, and the potential of genomic selection to contribute to disease control. An AGD challenge was performed in ∼1,500 Atlantic salmon, using gill damage and amoebic load as indicator traits for host resistance. Both traits are heritable (h^2^ ∼0.25-0.30) and show high positive correlation, indicating they may be good measurements of host resistance to AGD. While the genetic architecture of resistance appeared to be largely polygenic in nature, two regions on chromosome 18 showed suggestive association with both AGD resistance traits. Using a cross-validation approach, genomic prediction accuracy was up to 18% higher than that obtained using pedigree, and a reduction in marker density to ∼2,000 SNPs was sufficient to obtain accuracies similar to those obtained using the whole dataset. This study indicates that resistance to AGD is a suitable trait for genomic selection, and the addition of this trait to Atlantic salmon breeding programs can lead to more resistant stocks.

Salmonids are a high-value group of fish species, comprising 16.6% of global fish trade in 2013 (FAO 2016). Demand has grown steadily and is expanding geographically, and Atlantic salmon (*Salmo salar*) has the highest production volume and value of all the salmonid species (FAO 2016). However, in recent years, Atlantic salmon supply has fluctuated, partly as a result of infectious disease outbreaks in all major salmon producing countries (FAO 2017). These outbreaks are a major threat to sustainable production and future expansion of salmon aquaculture. While solutions to several bacterial and viral diseases (*e.g.*, vaccines) have been widely and routinely applied (Brudeseth *et al.* 2013), parasitic diseases are currently presenting a substantially greater problem to the industry. In addition to the major economic concern, these parasitic diseases and current treatment strategies can pose serious animal welfare and environmental concerns.

Amoebic gill disease (AGD), primarily caused by *Neoparamoeba perurans*, has been a perennial problem for salmon aquaculture in Australia, and outbreaks have become increasingly frequent in European salmon farms. It also affects other commercially important salmonids such as rainbow trout (*Oncorhynchus mykiss*) and chinook salmon (*Oncorhynchus tshawytscha*), and certain non-salmonid aquaculture species such as turbot (*Scophthalmus maximus*; Young *et al.* 2008). While gill disease symptoms are complex, AGD typically presents as multifocal white patches on the gill surface, lesions and epithelial hyperplasia leading to impaired gas exchange, poor growth and ultimately severe morbidity and mortality if untreated (Zilberg and Munday 2000; Adams and Nowak 2003). Current treatment strategies are crude, laborious, stressful to fish, and potentially environmentally damaging; for example involving hydrogen peroxide application or fresh water bathing of affected fish. This results in a large economic burden associated with the costs of treatment and productivity losses due to the disease. Therefore, alternative approaches that help control the impact of AGD are highly desirable.

One such method is improving the resistance of farmed salmon stocks to this disease via selective breeding, the benefits of which can be cumulative and permanent. Several studies have found significant estimates of heritability for disease resistance in aquaculture species (*e.g.*, Silverstein *et al.* 2009; Gjerde *et al.* 2011; Yáñez *et al.* 2014a; Palaiokostas *et al.* 2016, Tsai *et al.* 2016b). Harnessing this heritability for genetic improvement in selective breeding programs is a current goal. The high fecundity of aquaculture species, and resulting large full sibling family sizes, facilitates disease challenge testing of close relatives (*i.e.*, full siblings) to enable breeding value estimation in selection candidates. Selection is often more accurate when the relationship between individuals is obtained from genomic data (genomic selection) rather than the pedigree (traditional selection), but it depends on the architecture of the trait as well as other technical variables such as marker density (Daetwyler *et al.* 2010). For instance, genomic selection has been found to outperform traditional selection in resistance to sea lice in Atlantic salmon (Ødegard *et al.* 2014; Tsai *et al.* 2016; Correa *et al.* 2017b) and in resistance to pasteurellosis in sea bream (*Sparus aurata*; Palaiokostas *et al.* 2016). Further, while an initial study found no difference between genomic selection and pedigree-based approaches for resistance to bacterial cold water disease in rainbow trout (Vallejo *et al.* 2016), a later study with larger sample sizes resulted in doubling of accuracy with the genomic selection approach (Vallejo *et al.* 2017). One advantage of genomic selection over pedigree-based selection is that it more accurately captures the Mendelian sampling term between closely related individuals in the population—particularly relevant in aquaculture species with large families.

One of the main limitations of genomic selection is the cost; genotyping a large number of animals with a high density SNP panel could be prohibitive for all but the largest aquaculture breeding companies. Several strategies have been proposed to reduce the cost of genotyping for genomic selection in aquaculture via low density SNP panels, including within-family genomic selection (Lillehammer *et al.* 2013) and the use of genotyping strategies including imputation from low to high density SNPs (Kijas *et al.* 2016; Tsai *et al.* 2017). Genotype-by-sequencing technologies are also likely to help reduce costs in the near future given the continuously decreasing costs of sequencing and the advent of new sequencing technologies suitable for low to medium scale SNP genotyping, such as RAD-seq or GT-seq (Robledo *et al.* 2017). Reducing the cost of genomic selection will be critical to implement genomic selection in most aquaculture breeding programs, and in this sense improving the cost-effectiveness of genomic selection will likely be an important area of research in the coming years (Lillehammer *et al.* 2013).

Previous studies on host resistance to AGD in salmon have found estimates of heritability ranging from 0.16 to 0.48 (Taylor *et al.* 2007, 2009). The objectives of this study were a) estimate genetic variance of amoebic gill disease resistance in experimentally challenged Atlantic salmon, b) investigate the architecture of the trait using a single-SNP genome-wide association study (single-SNP GWAS) and regional heritability mapping, c) explore genomic selection using SNP markers and/or pedigree, and d) explore different marker densities with a view to future improvement of cost-effectiveness of genomic selection within commercial breeding programs.

## Materials And Methods

### Challenge experiments

An AGD challenge experiment using 1,481 Atlantic post-smolt salmon (∼18 months, mean weight ∼700 g) originating from a commercial breeding program (Landcatch, UK) was conducted by distributing the fish equally into 2 × 4 m seawater tanks in the experimental facilities of Machrihanish (Scotland, United Kingdom). Seeder fish with a uniform level of AGD infection were produced by cohabitation with infected fish from an *in vivo* culture. The challenge was then performed by cohabitation of infected seeder fish at a ratio of 15% seeder to naïve fish, allowing three separate cycles of infection with a treatment and recovery period after the first two (Taylor *et al.* 2009). For the first two challenges, fresh water treatment was performed 21 days after challenge, followed by a week of recovery. The disease was allowed to progress until the terminal sampling point in the third challenge. Fish were sampled and phenotypes were recorded during three consecutive days. A subjective gill lesion score of the order of severity ranging from 0 to 5 was recorded for both gills (Table 1; Taylor *et al.* 2016). These gill lesion scores were recorded by a single operator, who referred to pictures to guide classification. Some fish were scored by additional operators, and the scores never differed by >0.5. Further, one of the gills was stored in ethanol for qPCR analysis of amoebic load using *Neoparamoeba perurans* specific primers. Amoebic load has previously been used as a suitable indicator trait for resistance to AGD in salmon (Taylor *et al.* 2009). The challenged fish belonged to 312 different families with 1 to 37 fish per family. All fish were phenotyped for mean gill score (mean of the left gill and right gill scores) and amoebic load (qPCR values using *Neoparamoeba perurans* specific primers, amplified from one of the gills). All phenotypic information is available in File S1.

**Table 1:** Gill score description

**Gill score**	**Level of infection**	**Description**
0	Clear	Healthy red gills, no gross sign of infection.
1	Very light	One white spot, light scarring or undefined necrotic streaking
2	Light	2-3 spots/small mucus patch
3	Moderate	Established thickened mucus patches or spot groupings up to 20% of the total gill area
4	Advanced	Established lesions covering up to 50% of gill area
5	Heavy	Extensive lesions covering most of the gill surface

All animals were reared in accordance with relevant national and EU legislation concerning health and welfare. The challenge experiment was performed by the Marine Environmental Research Laboratory (Machrihanish, UK) under approval of the ethics review committee of the University of Stirling (Stirling, UK) and according to Home Office license requirements. Landcatch are accredited participants in the RSPCA Freedom Foods standard, the Scottish Salmon Producers Organization Code of Good Practice, and the EU Code-EFABAR Code of Good Practice for Farm Animal Breeding and Reproduction Organizations.

### Estimation of Amoebic load

Sampled whole gills were weighed and combined with an equal amount (wt/vol) 10 mM Tris, 1 mM EDTA, pH 8.0. Samples were then homogenized using a Qiagen TissueLyser II (Qiagen, Manchester, UK) following the manufacturers’ recommendations. Total DNA was extracted from 50 µl homogenate using Questgene 9600 DNA extraction kits (Questgene, York, UK) following manufacturers’ protocols. Amoebic load was determined via duplex qPCR reactions using primer/probe combinations targetting a 139 bp *N. perurans* specific 18S sequence (Fringuelli *et al.* 2012), and a 66 bp fragment of the Atlantic salmon Elongation Factor α 1 gene (Bruno *et al.* 2007). DNA was normalized to 50 ng/µl, and 5 µl was combined into 50 µl QPCR duplex reactions comprising: 1X Taqman QPCR reaction mix (Questgene, York, UK), 300 nM *N. perurans* specific primers, 150 nM *N. perurans* specific probe, 150 nM ELFα primers, and 75 nm ELFα probe (Table S1). Ampifications were performed using a Biorad iCycler iQ QPCR Detection System. The thermal profile consisted of 95° for 10 min and 45 cycles of 15 s denaturation at 95°/30 s annealing/extension at 56°. Fluorescence in both FAM and HEX channels was acquired during the annealing/extension stage. Ct (threshold cycle) values were recorded and the level of *N. perurans* load was normalized against the ELF internal control by computing the ratio Equivalent Target Amount (ETA) *N. perurans* : ETA ELFα.

### Genotyping

DNA was extracted from fin tissue samples using the DNeasy 96 tissue DNA extraction kit (Qiagen, UK) and samples were genotyped using an Illumina combined species Atlantic salmon and rainbow trout SNP array (∼17K SNPs, File S2), designed from a subset of SNPs from a higher density array (Houston *et al.* 2014). Genotypes (File S3) were filtered according to the following criteria: SNP call-rate < 0.9, individual call-rate < 0.9, FDR rate for high individual heterozygosity < 0.05, identity-by-state > 0.95 (both individuals removed), Hardy-Weinberg equilibrium FDR p-value < 0.05, minor allele frequency < 0.05. After this filtering, a total of 1,430 fish and 7,168 SNPs remained for further analysis. The large number of SNPs removed by filtering is due to the lack of informativeness of the rainbow trout SNPs in these Atlantic salmon samples.

### Estimation of genetic parameters

Gill score and gill qPCR data were analyzed using linear mixed models, fitting collection date (3 levels) and tank (2 levels) as fixed effects and animal as a random effect. The additive effect was estimated using both the genomic kinship matrix (**G**-matrix) and the pedigree (**A**-matrix). Heritabilities were estimated by ASReml 3.0 (Gilmour *et al.* 2014) fitting the following linear mixed model:

y=μ+Xb+Za+e

where **y** is a vector of observed phenotypes, *μ* is the overall mean of phenotype records, **b** is the vector of fixed effects of collection date and tank, **a** is a vector of additive genetic effects distributed as ∼N(0,**G***σ*^2^a) or N(0,**A***σ*^2^a) where *σ*^2^a is the additive (genetic) variance, **G** and **A** are the genomic and pedigree relationship matrices, respectively. **X** and **Z** are the corresponding incidence matrices for fixed and additive effects, respectively, and **e** is a vector of residuals. The genomic relationship matrix was constructed by the GenABEL R package (Aulchenko *et al.* 2007) using the method of VanRaden (VanRaden 2008) and then inverted by applying a standard R function. Phenotypic correlations between traits and genetic correlations were estimated using bivariate analyses implemented in ASReml 3.0 (Gilmour *et al.* 2014) fitting the linear mixed model described above.

### Single-SNP genome-wide association study

The single-SNP GWAS was performed using the GenABEL R package (Aulchenko *et al.* 2007) by applying the mmscore function (Chen and Abecasis 2007), which accounts for the relatedness between individuals applied through the genomic kinship matrix. Significance thresholds were calculated using a Bonferroni correction where genome-wide significance was defined as 0.05 divided by number of independently segregating SNPs (Duggal *et al.* 2008) and suggestive as one false positive per genome scan (1/number of independently segregating SNPs). The number of independently segregating SNPs was calculated using Plink v.1.9 (Chang *et al.* 2015) accounting for linkage disequilibrium among the consecutive SNPs. SNPs showing r^2^ values > 0.9 were considered linked.

### Regional heritability mapping

A regional heritability mapping (RHM) analysis (Nagamine *et al.* 2012; Uemoto *et al.* 2013) was performed where the genome was divided into overlapping regions consisting of 20 sequential SNPs and overlapping by 10 SNPs using Dissect v.1.12.0 (Canela-Xandri *et al.* 2015). The significance of the regional heritability for each window was evaluated using a log likelihood ratio test statistic (LRT) comparing the global model fitting all markers with the model only fitting SNPs in a specific genomic region (File S4). These windows overlap and therefore the significance threshold was determined using a Bonferroni correction using half the number of tested windows.

### Genomic prediction

The accuracy of genomic selection was estimated by five replicates of fivefold cross-validation analysis (training set 80%, validation set 20%). The phenotypes recorded in the validation population were masked and breeding values were estimated using ASReml 3.0 using the linear mixed model described above. Prediction accuracy was calculated as the correlation between the predicted EBVs of the validation set and the actual phenotypes divided by the square root of the heritability estimated in the validation population [*∼ r(y_1_* , *y_2_) / ^2^√h^2^* ]. Genomic best linear unbiased prediction (GBLUP) was applied to predict the masked phenotypes of the validation sets and the resulting prediction accuracy was compared to that of pedigree-based BLUP (PBLUP). The bias of the EBVs was estimated as the regression coefficient of the phenotypes on the predicted EBVs. Since medium-density SNP array genotyping can be expensive, we also evalutated the impact of reduced SNP density on prediction accuracy by using subsets of the SNP data for the GBLUP. To choose the SNPs for the (pseudo) low density panels we tried two different strategies: 1) we progressively increased the minimum allele frequency threshold in increments of 0.05 (maf, 0.05, 0.10, 0.15, …) resulting in genotype datasets with progresively lower SNP density and progressively higher MAF; and 2) we iteratively removed the SNP showing the lowest mean distance to the previous and the next SNP on the genome, resulting in datasets of evenly spaced genotypes.

### Data availability

Primers and probes to perform amoebic load estimation by qPCR are provided in Table S1. Phenotypic data of the fish used in this study is available in File S1. Note that gill scores correspond to an experimental challenge, gill scores higher than 0.5-1 are rarely encountered in Landcatch commercial facilities. Markers included in the SNP array and their position in the Atlantic salmon genome can be found in File S2. Genotypes of the fish used in this study are available in File S3. The regional heritability mapping model is detailed in File S4.

## Results And Discussion

The means and standard deviations for AGD resistance traits were 2.79 ± 0.85 and 31.36 ± 3.24 for the gill score and qPCR amoebic load, respectively. Moderate heritability estimates were observed for both phenotypes, which ranged between 0.25 and 0.36 (Table 2), and both the phenotypic and genetic correlations between the two traits were high and positive (0.81 and ∼1 respectively).

**Table 2:** Heritability estimates for the AGD resistance traits

	**Pedigree**	**gMatrix**
**Mean gill score**	0.25 ± 0.06	0.24 ± 0.04
**Amoebic load**	0.36 ± 0.07	0.25 ± 0.04

A previous study on AGD disease resistance within the Tasmanian Atlantic salmon population found similar heritability estimates, ranging from 0.16 for gross gill score (similar to mean gill score here) to 0.35 for digital image gill score (Taylor *et al.* 2007). Higher heritability estimates were obtained in the study of Taylor *et al.* (2009), which varied from 0.23 to 0.48 for mean gill score depending on the number of rounds of re-infection. The highest heritability, 0.48, corresponded to the third challenge trial after two rounds of infection and subsequent freshwater treatment, as in our study. This challenge model is based on results from Taylor *et al.* (2009) which showed that the gill scores from the third challenge is the most accurate predictor of ultimate survival, potentially implying genetic variation in the adaptive immune response.

Similar heritability estimates were obtained for host resistance to sea lice; ∼0.2 to 0.3 for the North Atlantic sea louse (*Lepeophtheirus salmonis*; Kolstad *et al.* 2005; Gjerde *et al.* 2011; Gharbi *et al.* 2015; Tsai *et al.* 2016), and 0.1-0.3 for the Pacific sea louse (*Caligus rogercresseyi*; Lhorente *et al.* 2012; Yáñez *et al.* 2014a; Correa *et al.* 2017a). Similarly, the heritability of resistance to *Gyrodactylus salaris*, another ectoparasite mainly affecting wild Atlantic salmon, was estimated to be 0.32 (Salte *et al.* 2010). These heritabilities are comparable to estimates for host resistance to bacterial and viral infections (Ødegård *et al.* 2011; Yáñez *et al.* 2014b), and imply that selective breeding for improved resistance to parasites in salmon is a plausible goal.

### Single-SNP genome-wide association analysis and regional heritability mapping

**Figure 1:**
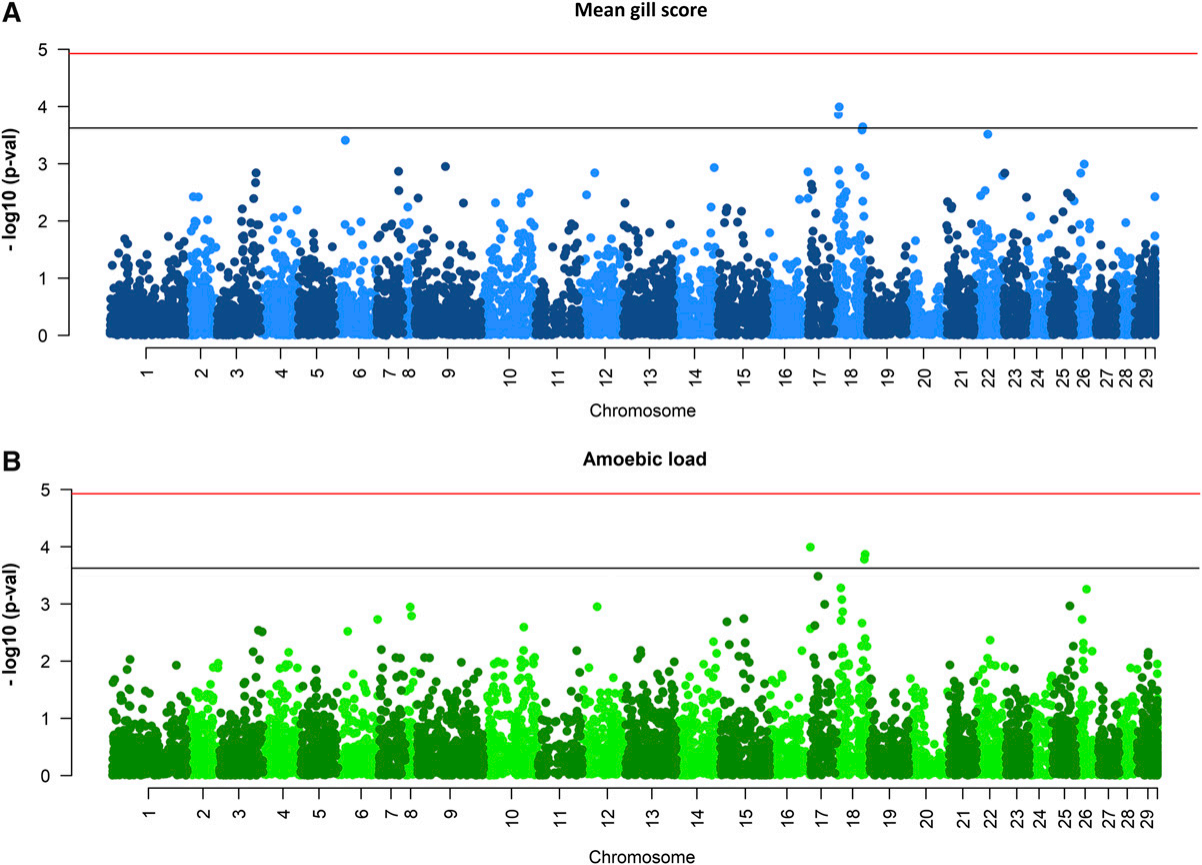
GWAS for resistance to AGD. Single-SNP GWAS results for A) mean gill score and B) amoebic load are shown. Horizontal bars represent Bonferroni corrected significance (red) and nominal signifcance (black).

The single-SNP GWAS revealed no major QTL regions that reached the genome-wide significance threshold for gill score or amoebic load (Figure 1). However, there were two suggestive QTL identified for both traits on chromosome 18, seemingly located in two non-overlapping regions around 9-12 Mb and 54-61 Mb respectively, each explaining ∼4% of the additive genetic variance (Table 3). The most significant SNP for amoebic load was observed at the distal end of chromosome 16. There were other genomic regions that either reach suggestive significance but only for one of the traits (*i.e.*, distal end of chromosome 16) or are close (chromosomes 6, 17 or 22), and these could also be QTL of moderate effect (∼3–4% of the additive genetic variance, Table 3) that might have been significant with a larger sample size.

**Table 3:** Top single-SNP GWAS markers for AGD resistance

**Mean gill score**				**Amoebic load**			
**Chr.**	**Position**	**Explained gen. var. (%)**	**p-val**	**Chr.**	**Position**	**Explained gen. var. (%)**	**p-val**
18	9,010,507	4.81	1.37E-04	16	87,305,577	4.40	1.03E-04
18	61,003,989	4.05	2.28E-04	18	61,003,989	4.17	1.37E-04
18	59,141,833	4.13	2.59E-04	18	59,141,833	4.21	1.67E-04
22	29,458,040	3.89	3.07E-04	17	17,603,968	3.98	3.30E-04
6	20,420,312	3.76	3.93E-04	18	9,010,507	3.82	5.30E-04
26	22,182,178	3.21	1.03E-03	26	22,182,178	3.41	5.60E-04
9	65,305,177	3.16	1.13E-03	18	11,619,560	3.20	8.43E-04
18	54,225,069	3.14	1.17E-03	17	31,447,688	3.19	1.03E-03
14	85,642,477	3.14	1.18E-03	25	37,782,067	3.05	1.10E-03
18	9,896,346	3.08	1.30E-03	12	31,597,392	3.06	1.13E-03
7	46,569,758	3.32	1.37E-03	8	13,396,576	3.17	1.14E-03
16	87,305,577	3.10	1.40E-03	18	13,403,715	2.99	1.39E-03
12	31,597,392	3.04	1.45E-03	8	49,527,638	2.85	1.63E-03
3	82,689,281	3.04	1.46E-03	15	49,527,638	2.79	1.82E-03
26	14,842,966	3.08	1.47E-03	6	83815992	2.77	1.88E-03

Chr.: chromosome; gen. var.: genetic variance.

**Figure 2:**
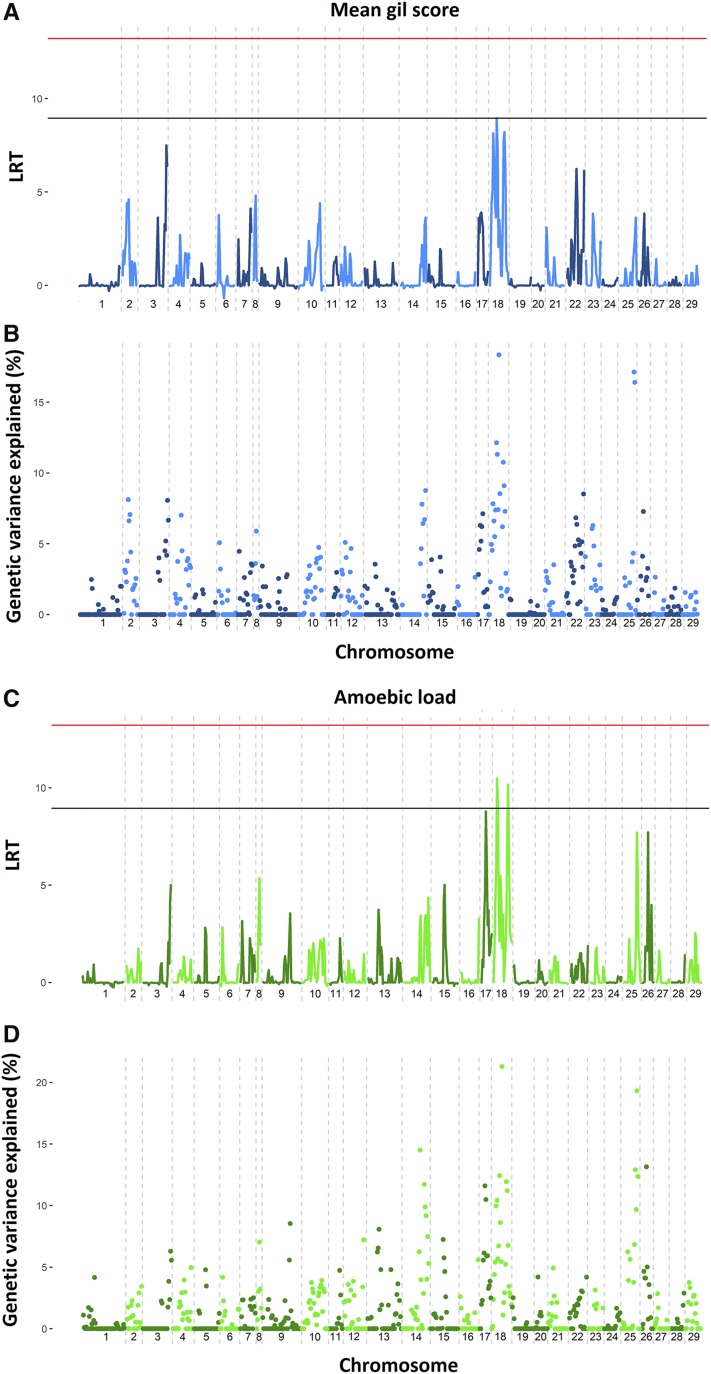
Regional heritability mapping for AGD resistance. Regional heritability mapping results for mean gill score and amoebic load are shown. A) and C) represent the log-ratio test values for each tested region (20 consecutive SNPs) for mean gill score and amoebic load respectively, horizontal bars represent Bonferroni corrected significance (red) and nominal significance (black). B) and D) represent the percentage of additive genetic variance explained by each region for mean gill score and amoebic load repectively.

The QTL identified by regional heritability mapping (RHM) were consistent with the results of the single-SNP GWAS, with two regions in chromosome 18 showing the highest significance for both mean gill score and amoebic load (Figure 2). These regions explained between 9.5 and 11.6% of the genetic variance respectively, and contained the most significant SNPs detected by the single-SNP GWAS. Another region in chromosome 18 between 25 and 42 Mb explained ∼20% of the heritability, but its significance was lower. The SNPs in this large region between the two putative QTL may be picking up on effects arising from either or both of the flanking regions due to linkage disequilibrium. Further, regions in chromosomes 17, 25 and 26 almost reached nominal significance for amoebic load, explaining >10% of the genetic variance. The most important discrepancy is in the distal region of chromosome 16, which shows no significant association in RHM but held the most significant marker in the amoebic load single-SNP GWAS. This difference might be explained by the high recombination rates found in the extremes of the chromosomes in Atlantic salmon (*e.g.*, Tsai *et al.* 2016a); the significant SNP was the penultimate marker in chromosome 16. RHM uses information from several consecutive markers, and has been shown to have an advantage over single-SNP GWAS to explain part of the typical missing heritability of single-SNP association studies and to detect QTL of small effects which otherwise would not be detected using information from single SNPs (Nagamine *et al.* 2012, Uemoto *et al.* 2013, Riggio and Pong-Wong 2014; Shirali *et al.* 2016).

Our results point toward a polygenic architecture of resistance to AGD, but potentially including a few QTL explaining moderate levels of the genetic variation. Genotyping additional AGD-challenged and phenotyped samples would help provide evidence in support or against the existance of these QTL. Further, a higher SNP density could possibly identify additional QTL not in linkage disequilibrium with the SNPs in this study, help to fine map the ones reported here, and possibly increase the estimates of genetic variation explained by the QTL.

While a few major disease resistance loci have been described, such as for viral infectious pancreatic necrosis in Atlantic salmon (Houston *et al.* 2008, Moen *et al.* 2009), the majority of disease resistance traits for aquaculture species are polygenic in nature (Houston 2017). Polygenic architecture has been observed for host resistance to sea lice (Tsai *et al.* 2016) and *Piscirickettsia salmonis* (Correa *et al.* 2015) in Atlantic salmon, pasteurellosis in gilthead sea bream (Palaiokostas *et al.* 2016) and *Gyridactylus salaris* in salmon (Gilbey *et al.* 2006). Other examples of putative major QTL include whirling disease in rainbow trout, caused by the myxosporean parasite *Myxobolus cerebralis*, which explains up to 86% of phenotypic variance depending on the family (Baerwald *et al.* 2011), bacterial cold water disease in trout where 27–61% of the genetic variation is explained by major QTL depending on the line (Vallejo *et al.* 2017), and Pancreas Disease in Atlantic salmon where approximately 20% of the genetic variation is explained by the largest QTL (Gonen *et al.* 2015). While resistance to parasitic disease does tend to show a polygenic architecture, and AGD is no exception, the putative QTL region(s) of moderate effect identified merit validation tests in independent populations, and functional genomic and resequencing studies to identify putative underlying genes and mechanisms.

### Genomic selection accuracy

Using a fivefold cross-validation analysis, the prediction accuracy with the genomic relationship (G) matrix was ∼18% higher than with the pedigree (A) matrix for both mean gill score and amoebic load, and GBLUP predictions showed practically no bias (Table 4). Prediction accuracies obtained for amoebic load measured by qPCR were ∼20% higher than those of mean gill score, which may be due to the wider range of the amoebic load trait. Taylor *et al.* (2007) found that gill damage scores obtained using image analysis or histopathology showed high positive genetic correlation, but correlation between these traits and gill score was lower. The prediction accuracy results from the current study suggest genomic selection will significantly outperform pedigree-based selection for AGD resistance, and that both gill score and qPCR measures of amoebic load are useful traits for selection for AGD resistance.

**Table 4:** Accuracy and bias of genomic selection

	**Pedigree**		**gMatrix**	
	**Accuracy**	**Bias**	**Accuracy**	**Bias**
**Mean gill score**	0.51	0.90	0.62	1.00
**Amoebic load**	0.60	0.88	0.70	0.99

**Figure 3:**
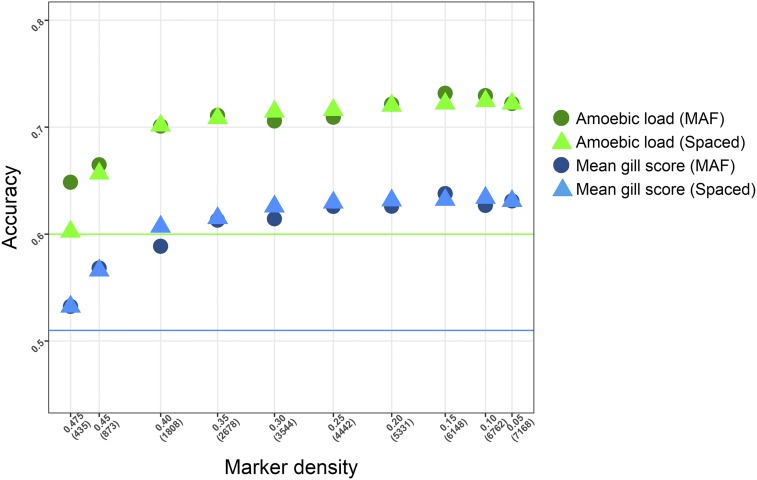
Prediction accuracy for different SNP densities. Accuracy of genomic prediction (GBLUP) for mean gill score and amoebic load with different SNP densities, selected based on their minimum allele frequencies (MAF) or their position in the genome so the markers are evenly spaced (Spaced). Horizontal lines indicate the accuracy of pedigree selection. X-axis figures represent MAF values and number of SNPs (in parenthesis).

Since genotyping with medium or high-density SNP arrays is relatively expensive, and aquaculture species tend to have closely related animals in training and validation populations (*e.g.*, in ‘sib testing’ schemes), well designed low density genotyping panels may be useful in genomic selection. When SNP density was reduced either via progressive increase in MAF thresholds or selecting evenly-spaced sets of markers, accuracy remained relatively stable until 1,808 SNPs where a gradual drop off in accuracy was observed (Figure 3). However, even at very low SNP density of 435 SNPs the accuracy of prediction was higher using GBLUP than PBLUP, except for Amoebic load estimated using the evenly spaced SNPs which resulted in an accuracy similar to that of PBLUP.

The results for genomic prediction of breeding values are generally consistent with published observations for aquaculture species to date. For host ressitance to sea lice, marked gains in accuracy were observed, from 10 to 52% depending on the population studied (Ødegard *et al.* 2014; Tsai *et al.* 2016); and recently different genomic selection models (ssGBLUP, wssGBLUP, BayesB) have been shown to almost double the prediction accuracy for bacterial cold water disease in rainbow trout compared to pedigree-based estimates (Vallejo *et al.* 2017). Interestingly, both studies on host resistance to sea lice in salmon showed practically no improvement in prediction accuracy when SNP density was increased above 5K (Ødegard *et al.* 2014; Tsai *et al.* 2016). As shown in the current study, genomic prediction accuracy is higher compared to pedigree-based prediction even when we use very low density genotyping (a few hundred SNPs). This is somewhat surprising given the size of the salmon genome (∼3 Gb, Lien *et al.* 2016), but probably reflects the close relationship between the training set and the reference set in the cross validation design – *i.e.*, full and half siblings will occur in both sets. The high accuracy with low marker density may also reflect aspects of the salmon population history, for example relatively low effective population size and past admixture may be expected to result in long-range LD and this may increase the predictive ability of a sparse SNP marker set.

Genotyping costs can be an important hurdle for the application of genomic selection, especially for small companies and breeding programs. For example, in mass spawning species that require genotyping to ascertain the pedigree, genomic selection could potentially be applied without a major genotyping cost increase. Further, this can be combined with genotyping strategies and imputation to improve cost-effectiveness, *e.g.*, Tsai *et al.* (2017) showed that imputation from 250 SNPs to ∼25K led to an improvement in prediction accuracy of 21% compared to pedigree prediction. Such strategies may increase cost-effectiveness and therefore uptake of genomic selection in aquaculture breeding, with beneficial impact on disease resistance and control.

### Conclusions

Host resistance to AGD in Atlantic salmon is moderately heritable (h^2^ ∼0.25 - 0.30) and can be measured using indicator traits such as gill score or amoebic load measured by qPCR. The genetic architecture of AGD resistance appears to be polygenic, but with two suggestive QTL explaining up to 11% of the genetic variance on chromosome 18, and other non-significant regions in other chromosomes accounting for a similar amount of variance. These possible QTL should be tested in independent populations, and may form the basis for identification of underlying causative genes. Genomic prediction accuracy was substantially higher (∼18%) when using genomic relationships rather than pedigree-based relationships with a ∼7K SNP panel, and remained so even when marker density substantially reduced. Since AGD is a large threat for salmon aquaculture in most major salmon production countries, genomic selection is likely to be an important component of breeding programs to help tackle this disease via genetic improvement of host resistance.

## Supplementary Material

Supplemental Material is available online at www.g3journal.org/lookup/suppl/doi:10.1534/g3.118.200075/-/DC1
