# Supplementary material for: Genome-Wide Association and Genomic Selection for Resistance to Amoebic Gill Disease in Atlantic Salmon
Source: G3 (Bethesda). 2018 Feb 2;8(4):1195–203. doi: 10.1534/g3.118.200075 (PMC5873910; doi:10.1534/g3.118.200075)
Supplement: Supplementary file 4 [file 1195FileS4.pptx]

## Slide 1
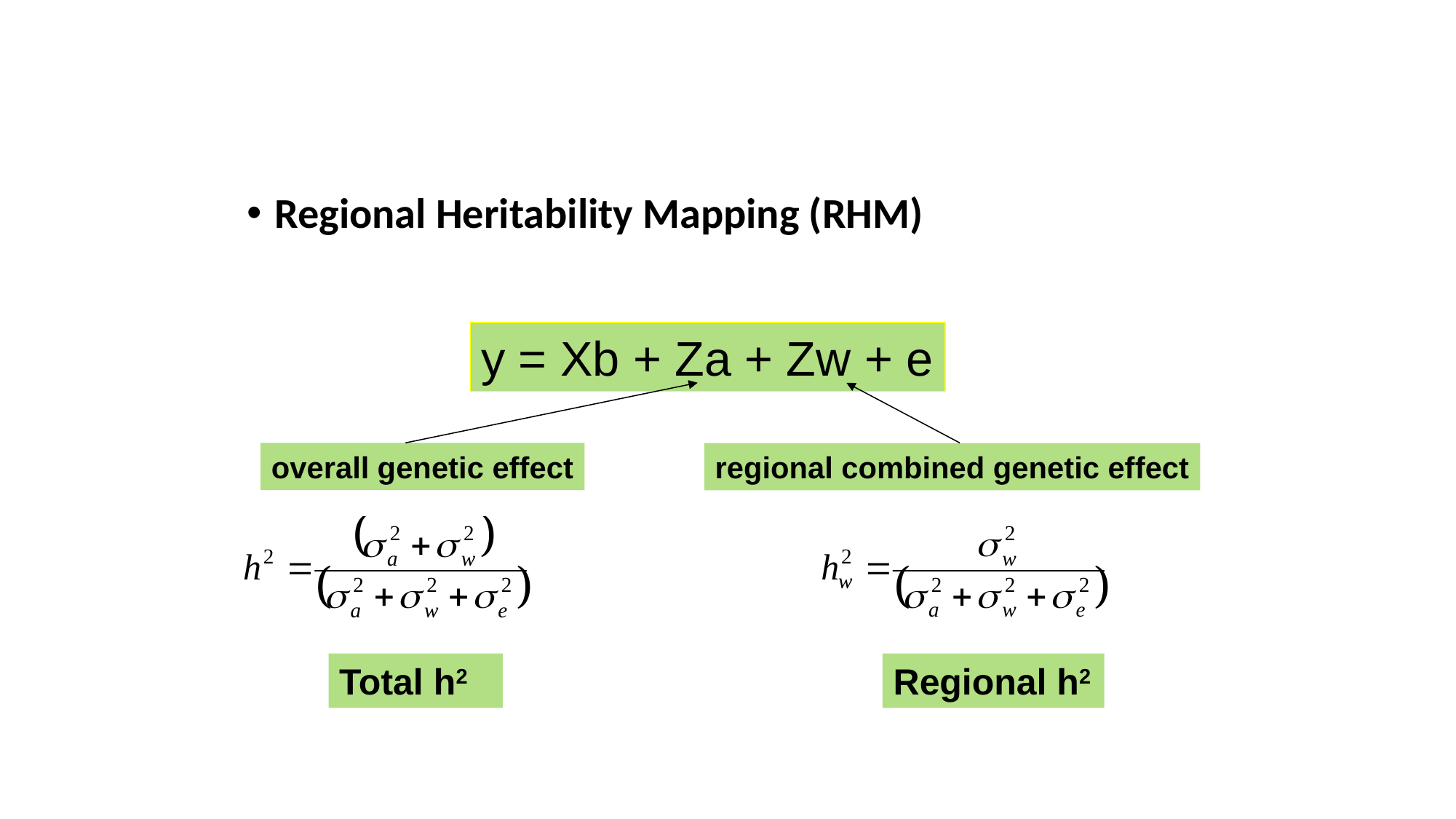

Regional Heritability Mapping (RHM)
y = Xb + Za + Zw + e
overall genetic effect
regional combined genetic effect
Total h2
Regional h2
