# Supplementary material for: Genome-Wide Association and Genomic Selection for Resistance to Amoebic Gill Disease in Atlantic Salmon
Source: G3 (Bethesda). 2018 Feb 2;8(4):1195–203. doi: 10.1534/g3.118.200075 (PMC5873910; doi:10.1534/g3.118.200075)
Supplement: Supplementary file 5 [file 1195TableS1.docx]

**Table S1. qPCR primers and probes for amoebic load estimation**

| **Sequence** | **Amplicon size (bp)** | **Reference** |
| --- | --- | --- |
| GTTCTTTCGGGAGCTGGGAG | 139 | Fringuelli *et al.*, 2012 |
| GAACTATCGCCGGCACAAAAG |  |  |
| 6-FAM-CAATGCCATTCTTTTCGGA_MGB |  |  |
| GGCCAGATCTCCCAGGGCTAT | 66 | Bruno *et al.*, 2007 |
| TGAACTTGCAGGCGATGTGA |  |  |
| HEX-CCTGTGCTGGATTGCCATACTG_MGB |  |  |

**References:** 1) Fringuelli, E., A. W. Gordon, H. Rodger, M. D. Welsh, and D. A. Graham, 2012 Detection of *Neoparamoeba perurans* by duplex quantitatitve Taqman real-time PCR in formalin-fixed, paraffin-embedded Atlantic salmonid gill tissues. J. Fish Dis. 35: 711-724. 2) Bruno, D., B. Collet, A. Turnbull, R. Kilburn, A. Walker, et al., 2007 Evaluation and development of diagnostic methods for *Renibacterium salmoninarum* causing bacterial kidney disease (BKD) in the UK. Aquaculture 269: 114-122.
